# Effects of Maternal Basking and Food Quantity during Gestation Provide Evidence for the Selective Advantage of Matrotrophy in a Viviparous Lizard

**DOI:** 10.1371/journal.pone.0041835

**Published:** 2012-07-26

**Authors:** Keisuke Itonaga, Susan M. Jones, Erik Wapstra

**Affiliations:** School of Zoology, University of Tasmania, Hobart, Tasmania, Australia; University of Texas Arlington, United States of America

## Abstract

The evolution of matrotrophy (i.e., direct supply of nutrients by the mother during gestation) may be associated with high maternal energy availability during gestation. However, we lack knowledge about the selective advantages of matrotrophic viviparity (live-bearing) in reptiles. In reptiles, the interaction between body temperature and food intake affect maternal net energy gain. In the present study, we examined the effects of basking and food availability (2 by 2 factorial design) during gestation on offspring phenotype in a matrotrophic viviparous lizard (*Pseudemoia entrecasteauxii*). Subsequently, we investigated if the maternal effects were context-dependent using offspring growth rate as an indicator of the adaptive significance of matrotrophy. Offspring were exposed either to the same thermal conditions as their mothers experienced or to thermal conditions different from those experienced by their mothers. We provide the first evidence that an interaction between maternal thermal and maternal food conditions during gestation strongly affects offspring phenotype, including date of birth, body size and performance ability, which affect offspring fitness. Offspring growth rate was dependent on offspring thermal conditions, but was not influenced by maternal effects or offspring sex. Matrotrophic viviparity provided gravid females with the means to enhance offspring fitness through greater energetic input to offspring when conditions allowed it (i.e., extended basking opportunity with high food availability). Therefore, we suggest that selective advantages of matrotrophic viviparity in *P. entrecasteauxii* may be associated with high maternal energy availability during gestation.

## Introduction

Maternal effects can include non-genetic effects that influence offspring phenotype. They are recognised as one of the most important components of evolutionary ecology [1]. Since the early 20^th^ century, a large number of studies have documented maternal effects in both plants and animals (e.g., insects: [2]; herbs: [3]; fish: [4]; reptiles: [5]). However, the adaptive significance of maternal effects is still an unresolved issue in evolutionary biology [6]–[8]. This is because maternal effects can occur without any important ecological implications [9], [10] or conversely may have positive or negative impacts on offspring fitness [6].

Despite the uncertain benefits of maternal effects, the evolution of viviparity (live-bearing) in squamate reptiles has been linked to maternal effects. Shine [11] suggested that the prolonged maternal control over embryonic developmental conditions in the uterus may provide selective advantages for offspring of viviparous reptiles (i.e., the Maternal Manipulation Hypothesis). Reptiles are ectothermic, so environmental conditions (especially temperature) strongly affect physiological processes. For example, embryonic temperature during embryogenesis has been shown to affect offspring phenotype in many species [12]–[14]. In viviparous reptiles, the temperature of embryonic development is controlled by maternal thermoregulation (reviewed in [15]), and this maternal control during gestation can enhance offspring fitness (e.g., [5], [16], [17]).

In viviparous animals, offspring fitness may involve a number of other maternal factors. For example, the magnitude of placental nutrient support during embryogenesis strongly affects offspring fitness in matrotrophic viviparous animals which utilise a source supplied by the mother to nourish embryos during pregnancy (reviewed in [18]). Indeed, Trexler and DeAngelis [19] predict that matrotrophic viviparity is more likely to evolve when food availability during gestation is high and constant. This model developed for fish has largely been ignored in studies of the evolution of viviparity and subsequent evolution of matrotrophy in reptiles. Past studies generally concentrated on the key selective force of maternal effects mediated through temperature [11], however maternal food availability may be important [20]–[22]. This is because in reptiles net energy gain is strongly associated with the interaction between body temperature and food intake [23], [24]. However, no study has investigated such interaction-induced selective advantages in matrotrophic viviparous reptiles.

Context-dependent or anticipatory maternal effects can enhance offspring fitness [6], [7]. They occur when there is a correlation between maternal and offspring environments, such that the reproducing females predict the offspring environment in response to their current environment and adjust offspring phenotype accordingly [6], [7], [25]. In reptiles, growth rate is an important component of offspring fitness [26]–[28], and benefits of growth rates can be dependent on the postnatal environment [29]. Therefore, offspring growth rate may be a reliable indicator as to whether mothers adjust offspring growth rate to postnatal environments. There is some evidence that maternal thermal conditions during gestation can influence offspring growth rate in viviparous reptiles [13], [30]. So, if context-dependent maternal effects have evolved in viviparous reptiles, it is possible that gravid females adjust offspring growth rate in response to maternal thermal (or other environmental) conditions, and that this enhances offspring, and their own, fitness.

In this study, we focused on how the interaction between maternal thermal and maternal food conditions (i.e., maternal net energy availability) during gestation affects offspring phenotype in a matrotrophic viviparous reptile. Matrotrophy may benefit offspring development (and fitness) when maternal net energy is high during gestation [19], [31], by enhancing fitness-related offspring phenotypic traits such as date of birth, body size, performance ability and amount of fat reserves [5], [20], [32]–[34]. Conversely, matrotrophy may be costly to offspring development (and fitness) when maternal net energy is low during gestation [35], [36]. In such situations, however, matrotrophic females may display strategies such as bet-hedging (i.e., variation in energy allocation among embryos) to prevent the costs of matrotrophy from outweighing its benefits in terms of maternal fitness [19], [37], [38].

We addressed these questions using southern grass skinks, *Pseudemoia entrecasteauxii*. They are small matrotrophic viviparous skinks [snout-vent length (SVL) 40–60 mm] and their nutrient provisioning for embryonic development is roughly half from the yolk and half via the placenta [21], [39]. This is an ideal model species to investigate the evolution of matrotrophic reproduction in reptiles; we have previously demonstrated the importance of transfer of nutrients, organic substances and hormones across the placenta [22], [40], [41] during embryo development, and here explore the importance of variation in two key environmental factors that affect female energy availability during gestation (temperature and food availability). This species is restricted to cold-temperate regions where food and thermal availability vary annually and seasonally [42]–[45]. In addition, females increase basking behaviour and maintain high feeding rates during gestation [22], [46] suggesting both maternal body temperature and energy gain are important to offspring development. We manipulated maternal basking (i.e., female body temperature) and maternal food availability (2 by 2 factorial design) during gestation and then measured date of birth, offspring body size, offspring performance ability, offspring fat reserves and variation in within-clutch offspring size (i.e., indicator of a bet-hedging strategy) to examine the effects of the interaction between maternal thermal and maternal food conditions during gestation on offspring. Subsequently, we measured offspring growth rate using reciprocal transplant experiments (manipulating thermal conditions only) to examine the potential for context-dependent maternal effects (e.g., [22], [47]). A similar design was used previously by Swain and Jones [32] to explore facultative versus obligate placentotrophy in a largely non-matrotrophic unrelated species (*Niveoscincus metallicus*); here we explore a larger question about the selective advantages of matrotrophic viviparity in reptiles.

## Results

### Maternal and offspring characteristics

There were no differences in initial maternal SVL among treatments (*F*_3, 159_ = 1.20, *P* = 0.3126) (Table 1). Although all females grew during gestation, maternal growth rate (mm/day) was significantly affected by the interaction between maternal basking and maternal food availability during gestation (Table 1 and 2). Post-hoc tests [Ryan-Einot-Gabriel-Welsch multiple range tests (REGWQ tests)] showed that females given extended basking with high food availability during gestation had a higher growth rate than females in other gestation conditions (Table 1). Although maternal growth rates varied between maternal treatments, there was no variation in postpartum maternal SVL between treatments (Table 1 and 2), presumably reflecting differences in gestation length between treatments (see below).

**Table 1:** Characteristics of maternal and offspring *Pseudemoia entrecasteauxii under four gestational regimes*.

		12 hours basking	12 hours basking	4 hours basking	4 hours basking
		+high food	+low food	+high food	+low food
Maternal characteristics	Total sample size	40	40	40	40
	Number of females giving birth	37	36	36	36
	Initial snout-vent length (mm)	44.93±0.76	46.26±0.90	46.04±0.72	44.92±0.43
	Postpartum snout-vent length (mm)	47.32±0.64	47.19±0.90	47.68±0.64	46.12±0.47
	Growth rate during gestation (mm/day)	0.054±0.005	0.019±0.003	0.016±0.002	0.011±0.001
	Postpartum body mass (g)	2.07±0.06	1.53±0.08	2.10±0.08	1.65±0.05
	Number of offspring	106	107	102	105
	Number of premature offspring	0	29	7	30
	Number of stillborn offspring	0	2	8	9
	Clutch size	2.84±0.17	2.94±0.20	2.83±0.16	2.92±0.18
	Relative clutch mass (unit-less: g/g)	0.26±0.01	0.30±0.02	0.22±0.01	0.26±0.01
	Within-clutch variation in offspring size (coefficient of variation)	5.59±0.02	7.28±0.79	6.93±1.14	9.00±1.10
Offspring characteristics	Snout-vent length (mm)	20.98±0.50	19.87±0.16	20.50±0.12	19.99±0.19
	Body mass (mg)	195.31±3.86	159.89±4.42	168.68±3.95	156.50±4.92
	Sprint speed (m s^−1^)	0.46±0.01	0.38±0.01	0.40±0.01	0.37±0.01
	Dry fat reserves relative to body mass (Student residual)	0.53±0.20 (35)	−0.40±0.08 (30)	0.01±0.21 (30)	−0.19±0.16 (30)

Characteristics of maternal *Pseudemoia entrecasteauxii* including snout-vent length, growth rate during gestation, body mass, clutch size, relative clutch mass and within-clutch variation in offspring size, and characteristics of offspring *Pseudemoia entrecasteauxii* from females given combinations of extended and restricted basking opportunities and food supply during gestation including snout-vent length, body mass, sprint speed and fat reserves relative to body mass at birth. Values are means ± S.E. (*n*).

**Table 2:** The results of statistical analyses for maternal and offspring characteristics.

	Variable	Basking condition	Food supply	Basking*Food
Maternal characteristics	Postpartum snout-vent length	*F*_1, 142_ = 0.03, *P* = 0.5868	*F*_1,__142_ = 1.67, *P* = 0.1989	*F*_1,__142_ = 1.60, *P* = 0.2076
	Postpartum body mass	*F*_1,__142_ = 2.83, *P* = 0.0948	***F*** **_1,__142_ = 54.31, ** ***P*** **<0.0001**	*F*_1,__142_ = 1.38, *P* = 0.2413
	Growth rate during gestation	***F*** **_1,142_ = 52.80, ** ***P*** **<0.0001**	***F*** **_1,142_ = 43.14, ** ***P*** **<0.0001**	***F*** **_1,142_ = 20.74, ** ***P*** **<0.0001**
	Clutch size	*F*_1,__142_ = 0.10, *P* = 0.7567	*F*_1,__142_ = 0.00, *P* = 0.9711	*F*_1,__142_ = 0.04, *P* = 0.8463
	Relative clutch mass	***F*** **_1,__128_ = 5.05, ** ***P*** ** = 0.0264**	***F*** **_1,__128_ = 7.73, ** ***P*** ** = 0.0063**	*F*_1,__128_ = 0.12, *P* = 0.7309
	Within-clutch variation in offspring size	*F*_1,__120_ = 2.30, *P* = 0.1317	***F*** **_1,__120_ = 7.09, ** ***P*** ** = 0.0088**	*F*_1,__120_ = 0.02, *P* = 0.8887
	Date of birth	***F*** **_1,__134_ = 18.41, ** ***P*** **<0.0001**	***F*** **_1,__134_ = 21.63, ** ***P*** **<0.0002**	***F*** **_1,__134_ = 11.44, ** ***P*** ** = 0.0009**
Offspring characteristics	Snout-vent length	*F*_1,__140_ = 2.30, *P* = 0.1313	***F*** **_1,__140_ = 27.24, ** ***P*** **<0.0001**	***F*** **_1,__140_ = 4.25, ** ***P*** ** = 0.0411**
	Body mass	***F*** **_1,__137_ = 15.80, ** ***P*** ** = 0.0001**	***F*** **_1,__137_ = 34.05, ** ***P*** **<0.0001**	***F*** **_1,__137_ = 7.98, ** ***P*** ** = 0.0054**
	Sprint speed	***F*** **_1,__145_ = 8.82, ** ***P*** ** = 0.0035**	***F*** **_1,__145_ = 17.29, ** ***P*** **<0.0001**	*F*_1,__145_ = 1.49, *P* = 0.2236
	Fat reserves relative to body mass	*F*_1,__121_ = 0.73, *P* = 0.3946	***F*** **_1,__121_ = 10.26, ** ***P*** ** = 0.0017**	***F*** **_1,__121_ = 4.29, ** ***P*** ** = 0.0405**

Summary of the results of statistical analyses of the effects of maternal basking and food availability (combinations of extended and restricted basking opportunities and food supply) and their interaction during gestation on female and offspring in *Pseudemoia entrecasteauxii*. Significant results are indicated in bold.

There was no effect of maternal basking availability during gestation and no interaction between maternal basking and maternal food availability during gestation on postpartum maternal body mass. However, the main effect of maternal food availability during gestation was significant (Table 2). Females with high food availability during gestation were heavier postpartum than females with low food availability (Table 1). Maternal treatments did not affect clutch size (Table 2). There was no interaction between maternal basking and maternal food availability on relative clutch mass (RCM), but the main effects of maternal basking and maternal food availability during gestation were significant (Table 2). Females given 12 hours basking during gestation showed significantly higher RCM than females given four hours basking during gestation. Similarly, females with low food availability during gestation showed significantly higher RCM than females with high food availability during gestation, presumably reflecting differences in maternal postpartum body mass (Table 1).

Maternal food availability during gestation significantly affected within-clutch variation in offspring size (i.e., CV), but maternal basking opportunity during gestation and the interaction between maternal basking and maternal food availability during gestation had no effect (Table 2). Within-clutch variation in offspring size was lower in females that had high food availability (6.22±0.60) than in females that had low food availability during gestation (8.14±0.68) (Table 1). Some females produced both fully-developed offspring and premature offspring in the same clutch. Six cases of this were observed in females given 12 hours basking with low food availability during gestation, four cases in females given four hours basking with high food availability during gestation, and seven cases in females given four hours basking with low food availability during gestation. Overall, we observed more premature offspring in females with low food availability during gestation (Table 1).

**Figure 1:**
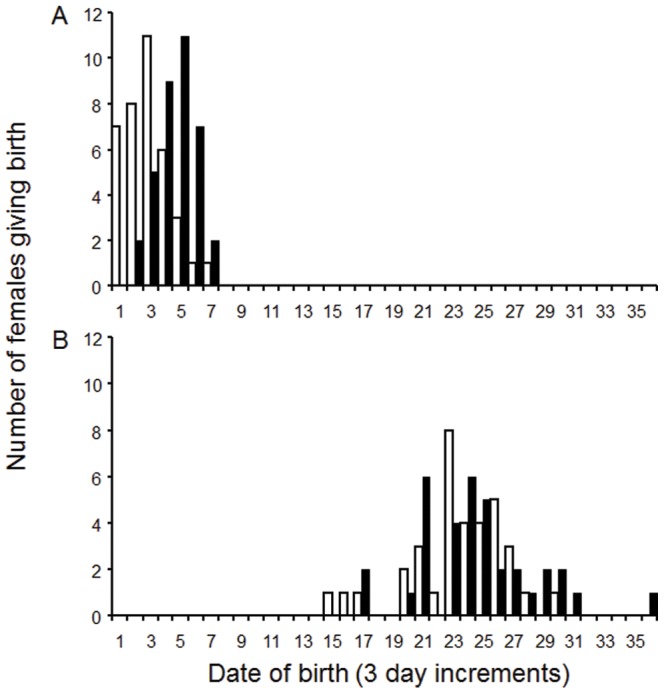
Frequency of births (3 day increments) for *Pseudemoia entrecasteauxii*. White bars indicate high food availability during gestation, while black bars indicate low food availability during gestation. Graph A shows date of birth in offspring from females given extended basking availability during gestation. Graph B shows date of birth in offspring from females given restricted basking availability during gestation. Three day increments begin with the first recorded births on Dec 7 2007.

Date of birth was affected by an interaction between maternal basking and maternal food availability during gestation (Table 2): REGWQ tests showed that females given 12 hours basking with high food availability during gestation gave birth about five days earlier than females given 12 hours basking with low food availability during gestation. Furthermore, mean birth date of all females in 12 hours basking treatments was about 55 days earlier than for females in four hours basking treatments (Fig. 1a and 1b).

**Figure 2:**
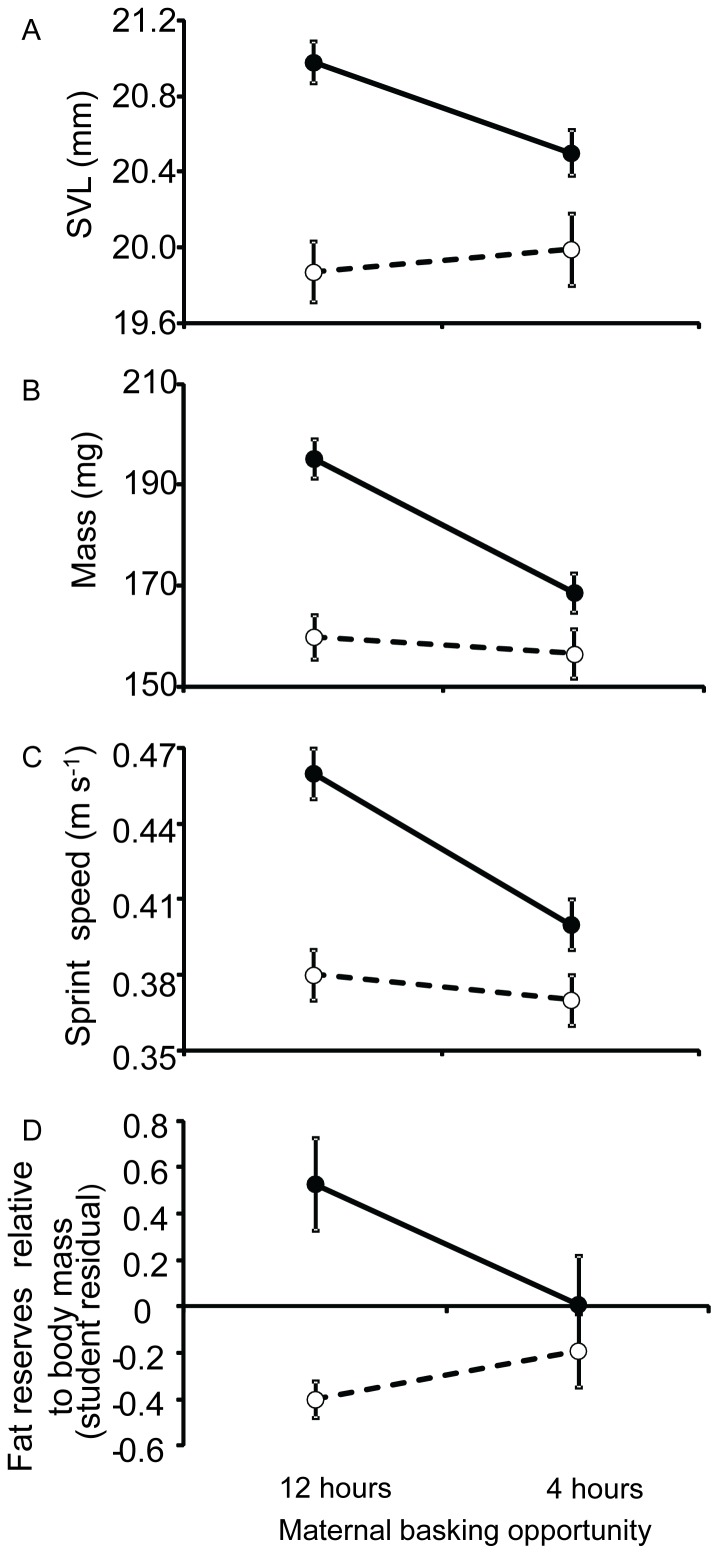
Effects of maternal gestation conditions on offspring *Pseudemoia entrecasteauxii*. A: snout-vent length (SVL), B: body mass, C: sprint speed and D: fat reserves relative to body mass at birth from females given combinations of extended and restricted basking opportunities and food supply during gestation. Values are means ± S.E. •: Offspring from females given high food availability during gestation. ○: Offspring from females given low food availability during gestation.

Most offspring phenotypic traits including SVL, mass and fat reserves were significantly affected by the interaction between maternal basking and maternal food availability during gestation (Table 2). REGWQ tests showed that females given 12 hours basking with high food availability during gestation produced offspring with larger SVL, heavier mass and larger fat reserves relative to body mass than females in other gestation conditions (Table 1 and Fig. 2a, 2b, 2d). Females given 4 hours basking with high food availability during gestation also produced offspring with larger SVL and heavier mass than females given 4 hours basking with low food availability during gestation or females given 12 hours basking with low food availability during gestation (Table 1 and Fig. 2a, 2b, 2d). Offspring sprint speed was influenced by both maternal basking and maternal food availability during gestation, but there was no interaction (Table 2). Offspring from females given 12 hours basking opportunities during gestation sprinted faster than offspring from females given four hour basking opportunities during gestation. Offspring from females with high food availability during gestation sprinted faster than offspring from females with low food availability during gestation (Table 1 and Fig. 2c).

### Assessing context-dependent maternal effects based on offspring growth rates

**Figure 3:**
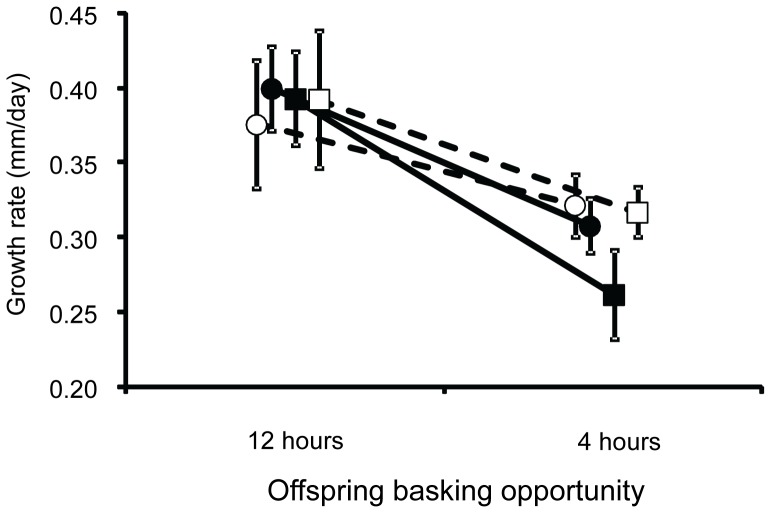
Offspring growth rate over five weeks in *Pseudemoia entrecasteauxii* under the two thermal regimes. •: Offspring from females given 12 hours basking opportunity with high food availability during gestation. ○: Offspring from females given 12 hours basking opportunity with low food availability during gestation. ▪: Offspring from females given 4 hours basking opportunity with high food availability during gestation. □: Offspring from females given 4 hours basking opportunity with low food availability during gestation. Sample sizes among offspring given 12 hours basking opportunity per day are • = 17, ○ = 13, ▪ = 16 and □ = 14. Sample sizes among offspring given 4 hours basking opportunity per day are • = 18, ○ = 17, ▪ = 13 and □ = 12. Values are means ± S.E. Some overlapping points have been displaced laterally to aid clarity.

Offspring growth rate was affected by offspring basking availability, but not by maternal gestation conditions, offspring sex or any interactions between maternal basking opportunity, maternal food availability, offspring basking opportunity and offspring sex (Table 3). Random choice of a single offspring for each clutch resulted in relatively even distribution of sexes among the treatments with no clear bias resulting from maternal treatments (27 males and 33 females from 12 hours of basking and 36 males and 24 females from 4 hours of basking). Offspring given 12 hours basking availability grew faster (0.056±0.043 mm day^−1^) than offspring given four hours basking availability (0.043±0.002 mm day^−1^) (Fig. 3).

**Table 3:** The results of statistical analyses for offspring growth rate.

*Source*	*F value*	*P value*
Maternal basking	*F*_1, 104_ = 0.00	*P* = 0.9804
Maternal food	*F*_1, 104_ = 0.01	*P* = 0.9059
**Offspring basking**	***F*** **_1, 104_ = 18.42**	***P*** **<0.0001**
Offspring sex	*F*_1, 104_ = 0.17	*P* = 0.6813
Maternal basking*maternal food	*F*_1, 104_ = 0.44	*P* = 0.5090
Maternal basking*offspring basking	*F*_1, 104_ = 0.00	*P* = 0.9936
Maternal basking*offspring sex	*F*_1, 104_ = 0.01	*P* = 0.9309
Maternal food*offspring basking	*F*_1, 104_ = 0.47	*P* = 0.4945
Maternal food*offspring sex	*F*_1, 104_ = 0.42	*P* = 0.5187
Offspring basking*offspring sex	*F*_1, 104_ = 0.03	*P* = 0.8554
Maternal basking*maternal food*offspring basking	*F*_1, 104_ = 0.00	*P* = 0.9770
Maternal basking*maternal food*offspring sex	*F*_1, 104_ = 1.70	*P* = 0.1951
Maternal basking*offspring food*offspring sex	*F*_1, 104_ = 0.01	*P* = 0.9044
Maternal food*offspring basking*offspring sex	*F*_1, 104_ = 0.89	*P* = 0.3474
Maternal basking*maternal food*offspring basking*offspring sex	*F*_1, 104_ = 0.94	*P* = 0.3347

Summary of the results of statistical analyses of the effects of maternal basking and food availability (combinations of extended and restricted basking opportunities and food supply) during gestation, offspring basking availability (extended and restricted basking opportunities), offspring sex and their interactions on offspring growth rates in *Pseudemoia entrecasteauxii*. The significant result is indicated in bold.

## Discussion

Our experiment provides the first evidence that maternal thermal conditions and maternal food conditions during gestation and importantly the interaction between them, strongly affect fitness related offspring phenotypic traits in a matrotrophic viviparous reptile. We showed that females given extended basking with high food availability during gestation produced offspring with earlier birth date, larger size, larger fat reserves relative to body mass, and faster sprint speed compared with females given other treatments. These effects may be explained by an acceleration of embryonic development due to increased maternal body temperature through extended basking opportunity [13], [48], [49], combined with increased net energy gain when food is abundant [23], [24], providing for enhanced nutrient transport across the placenta to developing embryos. High nutrient support for embryonic development via the placenta may increase offspring body size and body condition involving an increase in both muscle [50] and fat reserves [51]; see Thompson and Stewart [52] for related arguments.

 Date of hatching or birth, body size and locomotor performance ability are known to be important determinants of offspring fitness in a variety of taxa including reptiles (e.g., [5], [33], [53]–[57]). The potential fitness benefits related to offspring phenotype, such as early birth, large body size and high locomotor performance in reptiles include a higher survival over winter [56], [58], a higher early-age survivorship [59], [60], a higher predator avoidance [61], [62] and a larger size at maturation [12], [34]. Specifically, these effects are important to *P. entrecasteauxii* because their predator avoidance strategy is primarily sprinting [63], [64] and because adult body size in both sexes is positively associated with reproductive success (i.e., courtship and fecundity) ([65]; in the present study: maternal initial SVL and clutch size showed a positive relationship, *r* = 0.29, *P*<0.0001). Therefore, if offspring size at birth is positively correlated with size at a later date and importantly at maturation as it is in other small Australian lizards [5], [14], [34], [66], large offspring size at birth has an advantage in terms of future reproduction. However, large offspring size does not always have positive fitness effects; it can reduce survival rate when postnatal food is limited [29].We found that large offspring from females given extended basking with high food availability during gestation had large fat reserves relative to body mass. Even when feeding opportunities are limited in postnatal environments, large fat reserves may help these offspring to meet immediate crises. Thus, we suggest that high maternal net energy availability, (i.e., extended basking and abundant food during gestation) results in offspring with phenotypic traits such as early birth date, large body size, large fat reserves and fast sprint speed which may confer significant fitness benefits in *P. entrecasteauxii* (see also [40] for the importance of the quality of the female diet).

By contrast, when gestation conditions were unfavourable (i.e., restricted basking opportunity and especially limited food), we found that *P. entrecasteauxii* produced premature offspring and offspring with delayed birth, small size, low fat reserves relative to body mass and/or slow sprint speed. Such gestation conditions are described as low net energy gain conditions [24]. Furthermore, these offspring phenotypes have been shown to reduce survival and subsequent reproductive success in other reptiles (e.g., [33], [67]), and could be particularly important in our species if accompanied by reduced transport of crucial nutrients and organic substances such as leucine [21] and carotene [40] across the placenta to embryos. Therefore, we suggest that low net energy gain in females during gestation may increase the costs of matrotrophy in part because females may favour allocation of limited resources to their own needs including future reproduction rather than offspring [22].

We thus suggest that the fitness costs and benefits of matrotrophy in *P. entrecasteauxii* may depend on maternal net energy availability during gestation (*sensu* [19]), along with nutrient availability. The advantage of matrotrophy is that it can enable an adjustment of inputs to offspring when conditions allow. Therefore, we suggest that predictably high energy availability during gestation (especially in comparison to energy availability and predictability pre-gestation) may lead to consistent advantages of placental nutrient supply. In time, this may have been an important selective force for the evolution of matrotrophy and its associated mechanisms, in a similar way to which current thermal effects on offspring have been used to argue for viviparity [5], [16], [17]. In *P. entrecasteauxii*, as in other cold-temperate-zone viviparous reptiles, vitellogenesis occurs in autumn and early spring (e.g., [44], [68], [69]) when food availability is relatively low and unpredictable compared with the gestational periods of *P. entrecasteauxii* (i.e., late-spring to summer). One way to counter the costs on offspring fitness of matrotrophy when conditions are unfavourable is to alter energy allocation [19], [38]. We found that *P. entrecasteauxii* displayed a selective energy allocation among embryos and produced some better offspring within a single clutch at the sacrifice of fitness of other siblings when energy availability was limited during gestation.

So while we have demonstrated the benefits of matrotrophy in some situations (e.g., high food condition during gestation), why it has only evolved in a limited number of viviparous reptiles worldwide (i.e., 4 to 6 origins: [70]) is a key question. A possible explanation for the restricted number of evolutionary transitions to matrotrophy is that the costs of matrotrophy outweigh benefits in many viviparous species. For example, *P. entrecasteauxii* is one of a limited number of species which demonstrate an increase in basking and foraging to enhance maternal energy (and potentially nutrient and organic substance) gain during gestation [23], [24], [40], [46] presumably to support embryonic nutritional requirements. However, these behaviours can increase predation risks, especially for gravid females [46], [71]. Therefore, it is possible that the costs of such behaviours can outweigh the benefits in some viviparous reptiles. Furthermore, unpredictable food levels during gestation usually favour lecithotrophic reproduction (i.e., supply of nutrients by the yolk) [72], [73]. Another possibility is that placental structure limits nutrient transfer. In reptiles, viviparity has about 100 independent origins [74] with a variety of placental support and complexity [74]–[76]. Therefore, some evolutionary origins of placental structure may not have the potential for further evolution of matrotrophic reproduction.

Despite the effects of gestation conditions on offspring phenotype and the putative links to fitness in the wild, we found that postnatal conditions significantly affected offspring growth while maternal gestation conditions did not (i.e., we found no evidence for context-dependent maternal effects). In addition, offspring sex did not affect their growth which suggests that context-dependent sex allocation may not occur in this species in response to thermal or food conditions [25], [77]. There was no variation in growth rate among offspring in the same thermal regimes, even though their maternal gestation conditions were different. Thus, the maternal effects we demonstrated do not necessarily translate to effects on offspring growth (see also [47], [78]). Similarly, incubation temperature in Western fence lizard (*Sceloporus occidentalis*) strongly affected offspring morphology, but did not influence offspring growth rates [27], [79]. Mainwaring et al. [80] also found that in a passerine bird (*Cyanistes caeruleus*) postnatal environmental effects were more important than maternal effects in determining offspring growth rate. In addition, context-dependent maternal effects are only predicted under a specific set of circumstances (notably that females or offspring have apriori knowledge during development of the condition they will experience postnatally [6], [7]; for our species it is possible that the thermal conditions experience by females while gravid are not strongly reflected in the conditions their offspring experience.

In conclusion, we have provided the first demonstration that two key environmental factors (temperature and food availability) interact to influence offspring phenotype in a matrotrophic viviparous reptile. Matrotrophy allows maternal effects on offspring phenotype to respond to more than one environmental factor [20], [22], in this case basking opportunity and/or food intake during gestation. Trade-offs between fitness costs and benefits related to maternal net energy availability during gestation may have been an important determinant for the evolution of matrotrophy in *P. entrecasteauxii*. Thus, we suggest that matrotrophy may have evolved as a response to high net energy availability during gestation, which may enhance offspring fitness throughout maternal effects.

## Materials and Methods

### Maternal conditions during gestation

One hundred and sixty female *P. entrecasteauxii* were collected from one population at the Peter Murrell Reserve in Kingston, southern Tasmania, Australia (41°50′S, 146°36′E) between 15 and 31 October 2007, shortly after ovulation took place. We do not know the history of these females and recognise that their nutritional and thermal history may influence the expression of maternal effects, including for example transgenerational maternal effects. However, given that all females were collected from the same population over a restricted temporal window and because our design split females randomly into their treatments, we do believe this to be an important source of error. The lizards were taken to the Herpetology Laboratory at the School of Zoology at The University of Tasmania where they were measured SVL (±0.01 mm) and weighed (±1 mg). The presence of ovulated follicles was confirmed by palpation of the female's abdomen. Females were randomly assigned to one of the following four treatment groups (2 by 2 factorial design):

1. 12 hours basking opportunity per day with high food availability (three mealworms per lizard, three times per week);
2. 12 hours basking opportunity per day with low food availability (two mealworms per lizard, once a week);
3. four hours basking opportunity per day with high food availability; and
4. four hours basking opportunity per day with low food availability.

The thermal conditions reflect the annual variation in hours of sunlight typically encountered by Tasmanian skinks in the natural population and has been used by us for other reptile species to realistically vary thermal opportunity (e.g., [5], [25], [45], [81], [82]). The amount of food (high and low) per lizard during gestation was based on our standard husbandry conditions for this and similar sized skinks (i.e., six mealworms per lizard per week). Over long experience (e.g., [5], [25], [32]), we find that females maintained on such a diet remain healthy, do not lose weight nor gain excessive fat stores. We therefore both increased and decreased this amount of food to provide a comparatively high and low level of food. We recognize that variation in the frequency of feeding may affect female metabolism [83], but given the speed of digestion and that prey are not always consumed immediately, we do not believe that this introduces confounding effects in our analyses. They were maintained in an air-conditioned room under bright fluorescent tube lighting (≈20000 lux) and UV lighting (14L: 10D). Each lizard was housed individually in a plastic terrarium (length: 300 mm, width: 200 mm, depth: 100 mm) which contained paper pellets as a substrate, one terracotta saucer and one wooden block as basking sites, and one plastic plate as a shelter; water, supplemented with multi-vitamins and calcium, was available *ad libitum*. Basking heat was supplied by a 25-w spotlight positioned≈80 mm above a basking surface and the thermal gradient in each plastic terrarium was 12–40°C, while the basking light was on allowing free thermoregulation during this time. All lizards were maintained in these conditions until parturition. Each plastic terrarium was positioned randomly within the experimental group, and was repositioned weekly to minimize position effects.

### Maternal and offspring characteristics

For each female, the following data were recorded: date of birth (birthing synchrony), postpartum SVL, maternal growth rate during gestation, postpartum mass, clutch size, RCM and variation in offspring size within-clutch. Gestation length was estimated by assuming that all females ovulated on Nov. 1. Growth rate during gestation was calculated as (postpartum SVL - initial SVL) (gestation length)^−1^. Relative clutch mass was calculated as (total offspring mass) (female postpartum body mass immediately after parturition)^−1^ [46], therefore, RCM is unit-less (g per g). Variation in offspring size within-clutch was measured using the coefficient of variation (CV) [84]. The range of clutch sizes was one to six; however, only 10 females produced a single offspring: we removed those offspring from the data set for the CV. The CV expresses variability in within-clutch offspring size relative to the magnitude of the mean within-clutch offspring size. The estimates of within-clutch variation in offspring size are not affected by differences in clutch size among females [85].

For all offspring, we recorded whether they were born alive or dead, SVL, body mass, body pigmentation and sprint speed as a measure of whole body performance [13] on the day of birth. Offspring were not sexed at birth because hemipenes eversion as a predictor of gonadal sex has not been verified in this species and moreover offspring are very small at birth (less than 200 mg) and we did not want to risk tail loss (or stress) prior to growth experiments (see below). Offspring with a weakly pigmented body (transparent body colour and/or red chest colour) were defined as “premature” offspring. Prior to the sprint trials, each lizard was held in a petri dish in a water bath (28±1°C) for 30 min to reach the optimal temperature for sprinting for this species [86]. Sprint time along the track (120 cm long and 8 cm wide) was recorded by five equally spaced (20 cm) infrared light beams. The fastest sprint speed over a 20 cm distance was taken as the maximum sprint speed (e.g., [63]). Lizards were encouraged to run by occasional gentle taps on the tail using a soft paint brush.

In clutches of two or more offspring, one offspring was selected randomly and killed on the day of birth to determine dry fat reserves. *Pseudemoia entrecasteauxii* stores fat as both abdominal fat bodies and in caudal fat stores. To estimate the mass of abdominal fat bodies for each offspring, the abdominal fat bodies were dissected out and transferred to a pre-weighed (±0.1 mg) eppendorf tube. The abdominal fat bodies were dried in a 60°C oven at least for two days, and re-weighed (±0.1 mg). To estimate the mass of caudal fat storage for each sample, we used the method of Chapple and Swain [87], modifying the volume of diethyl ether for the small size of the offspring. The tail was removed at the highest fracture plane, cut into small pieces and transferred to a pre-weighed (±0.1 mg) eppendorf tube. The tail was then dried in a 60°C oven for at least for two days and the tube was reweighed (±0.1 mg). The dried tail mass was calculated as (dried tail plus tube mass – tube mass). The dried tail was then immersed in diethyl ether (1 ml) for at least two days to dissolve the lipid stores, and then transferred to fresh pre-weighed (±0.1 mg) eppendorf tubes, and placed in a fume cabinet overnight to allow the diethyl ether to evaporate completely. The tube was then re-weighed (±0.1 mg), to determine the mass of tail minus fat.

### Assessing context-dependent maternal effects based on offspring growth rates

We measured offspring growth rate using reciprocal transplant experiments (manipulating thermal condition only). We randomly selected one offspring per litter from each maternal treatment. The rest of the offspring was released in the field. Each offspring was given a unique toe clip for permanent identification. These offspring were allocated into one of two conditions, either 12 hours or four hours basking opportunity per day. This means that the thermal conditions for half of the offspring were identical to those their mothers had experienced during gestation, while the other half of the offspring received a different thermal treatment. Up to five individuals were housed in a plastic terrarium (length: 300 mm, width: 200 mm, depth: 100 mm). Each cage contained paper pellets as a substrate, two terracotta saucers and one wooden block as basking sites, and one plastic plate as a shelter. Water, supplemented with multi-vitamins, was available *ad libitum*. Lizards were fed human baby food (HEINZ® pear flavour), supplemented with multi-vitamins, calcium powder and protein powder three times per week. Other conditions were as described for the females. We measured offspring SVL weekly for five weeks (e.g., [13], [22], [47]). The growth rate of each individual was calculated using the slope of the least squares regression line of offspring SVL against time (in weeks). In addition, at the end of growth experiment, we determined offspring sex by eversion of hemipenes to investigate if sex had an influence on growth rate and to confirm there were no strong sex biases resulting from maternal treatments.

### Statistical analyses

All statistical analyses were performed with SAS ® 9.1 for Windows. Differences in maternal initial SVL between maternal treatments were examined using one-way ANOVA. Influences of maternal gestation conditions (i.e., basking and food availability) and their interaction on maternal characteristics, including the date of birth, postpartum SVL, growth rate during gestation, postpartum body mass, clutch size, RCM and within-clutch variation in offspring size (CVs) were examined using full model two-way ANOVA. Basking (12 hours, four hours) and food availability (high, low) were considered as fixed factors. Assumptions of normality were checked by examining plots of standardized residuals against estimated values and the normal probability curve of the residuals; consequently the data for postpartum mass, clutch size and CV were log transformed, and the data for growth rate during gestation and date of birth were square root transformed to meet the assumptions of ANOVA.

Influences of maternal gestation conditions (i.e., basking and food availability) and their interaction on offspring phenotype, including SVL, body mass and sprint speed, were examined using full mixed-model ANOVA. In the mixed-model ANOVA, maternal identity was treated as a random factor to account for litter/clutch effects. Fat reserves were examined using full model two-way ANOVA. To examine differences in offspring fat reserves, we used residuals (i.e., fat reserves relative to body mass), which were generated from a regression analysis of all values for offspring fat reserves (abdominal fat bodies plus caudal fat storage) against offspring body mass. This is because, in general, body mass and fat reserves are positively correlated [88]. In the present study, this assumption was supported overall (*r* ^2^ = 0.4371, *P*<0.0001) and also in each maternal treatment. Maternal basking and maternal food availability during gestation were considered as fixed factors. Data for offspring sprint were log transformed to meet the assumptions of ANOVA.

To investigate context-dependent maternal effects, we used a full model four-way ANOVA to analyse the effect of maternal basking and maternal food availability during gestation, offspring basking availability and offspring sex and their interactions on offspring growth rate. Maternal basking and maternal food availability during gestation and offspring basking availability and offspring sex were considered as fixed factors.

All research was carried out with approval of the Animal Ethics Committee (A0009687) of the University of Tasmania, and a permit from the Department of Primary Industries, Water and Environment (FA07177).
